# Accuracy of percutaneous pedicle screw placement with 3-dimensional fluoroscopy-based navigation: Lateral decubitus position versus prone position

**DOI:** 10.1097/MD.0000000000033451

**Published:** 2022-04-07

**Authors:** Ryuichiro Okuda, Hisanori Ikuma, Tomohiro Inoue, Masataka Ueda, Tomohiko Hirose, Kazutoshi Otsuka, Keisuke Kawasaki

**Affiliations:** a Department of Orthopaedics Surgery, Kochi Health Sciences Center, Kochi, Japan; b Department of Orthopaedic Surgery, Kagawa Prefectural Central Hospital, Takamatsu, Japan; c Otsuka Orthopaedic Clinic, Takamatsu, Japan.

**Keywords:** 3D fluoroscopy-based navigation system, accuracy, downside PPS, lateral decubitus position, percutaneous pedicle screw, PPS deviation, PPS insertion, prone position, single-position spine surgery, upside PPS

## Abstract

The accuracy of percutaneous pedicle screw (PSS) placement in the lateral decubitus position has seldom been reported. This study aimed to retrospectively compare the accuracy of PPS placement with 3-dimensional (3D) fluoroscopy-based navigation in 2 cohorts of patients who underwent surgery in the lateral decubitus or prone positions at our single institute. A total of 265 consecutive patients underwent spinal surgery with PPS from T1 (thoracic 1) to S (sacrum) under the 3D fluoroscopy-based navigation system at our institute. Patients were divided into 2 groups based on their intraoperative patient positioning: lateral decubitus (Group L) or prone (Group P). A total of 1816 PPSs were placed from T1 to S, and 76 (4.18%) PPSs were assessed as deviated PPS. Twenty-one of 453 (4.64%) PPSs in Group L deviation and 55 of 1363 (4.04%) PPSs in Group P had deviated PPS, but with not significant difference (*P* = .580). In Group L, although the PPS deviation rate was not significantly different between the upside and downside PPS, the downside PPS significantly deviated toward the lateral side compared with the upside PPS. The safety and efficacy of PPS insertion in the lateral decubitus position were similar to those in the conventional prone position.

**Key Points**

- When the 3D fluoroscopy-based navigation system was utilized for PPS insertion, there was no significant difference in PPS deviation regardless of the intraoperative patient positioning (lateral decubitus position vs prone position).
- Our results suggest that the safety and efficacy of PPS insertion in the lateral decubitus position are similar to those in the conventional prone position. Moreover, surgeons should keep in mind that it is easy to deviate toward the lateral side of the downside PPS.

## 1. Introduction

Posterior spinal interbody fusion with instrumentation using percutaneous pedicle screws (PPS) is becoming a standard surgical procedure for thoracolumbar pathology. Accurate PPS placement is critical to avoid permanent neurological symptoms and the subsequent worsening of the patient health-related quality of life.^[1,2]^

Lateral interbody fusion and PPS (LIF-PPS) fixation require intraoperative repositioning to allow for easier PPS insertion. Studies on single-position LIF-PPS fixation have been emerging,^[3–6]^ but the accuracy of the procedure has not been reported extensively. Moreover, investigations on single-position LIF-PPS using O-arm-based navigation have reported benefits, such as reduced operating room occupancy time and reduced workforce requirements.^[4]^

The accuracy of PPS placement in the prone position has improved with the introduction of an intraoperative 3-dimensional (3D) navigation system over the last decade; however, the accuracy of PSS placement in the lateral decubitus position has seldom been reported.^[4,6]^

This study aimed to retrospectively compare the accuracy of PPS placement with 3D fluoroscopy-based navigation in 2 cohorts of patients who underwent surgery in the lateral decubitus or prone positions at our single institute.

## 2. Materials and methods

This single-center retrospective study was conducted at the in Kagawa Prefectural Central Hospital, Japan. The study protocol was reviewed and approved by the Committee on Ethics and Institutional Review Board of the Kagawa Prefectural Central Hospital (No.1037). The informed consent was obtained from all patients using the opt-out method.

### 2.1. Patient selection

This study included patients who underwent posterior instrumentation with PPS ranging from thoracic 1 (T1) to S, using a 3D fluoroscopy-based navigation system between April 2016 and March 2020.

Patient data were collected from medical records and analyzed, including patient-specific characteristics (age, sex, body mass index [BMI], and diagnosis), treatment-related characteristics (intraoperative position, number of PPS used, level of PPS inserted, and the position of reference frame), and PPS-specific characteristics (Ravi scale grades [Grade I: no deviation, Grade II: <2 mm, Grade III: 2–4 mm, Grade IV: >4 mm] and the direction of PPS deviation).^[7]^

Patients were divided into 2 groups according to the intraoperative position as follows: lateral decubitus position (Group L) and prone position (Group P). The surgical position was determined based on the diagnosis, surgical procedure, and surgeon preference. The distance between the level of PPS insertion and the position of the reference frame was assessed to verify the accuracy of the navigation system. In Group L, the PPS was divided into upside and downside PPS.

### 2.2. Surgical technique, patient position, and 3D fluoroscopy-based navigation system

Three well-trained spinal surgeons were involved in this study. The distribution of surgeons in each group was the same. None of the trainees were involved in this study.

**Figure 1.**
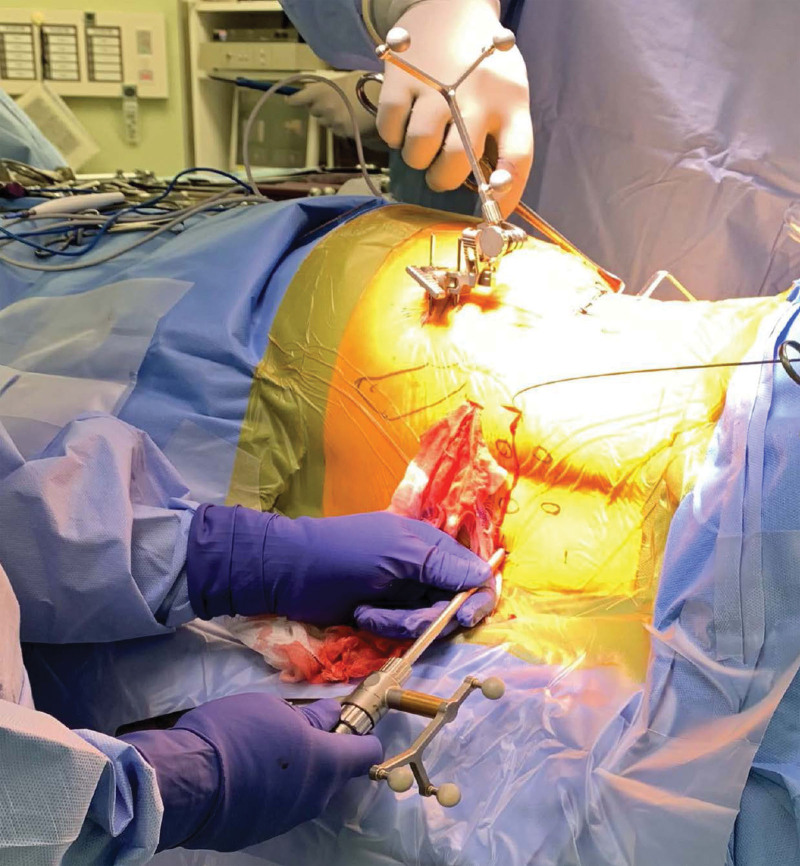
A guide wire is inserted through the target device.

**Figure 2.**
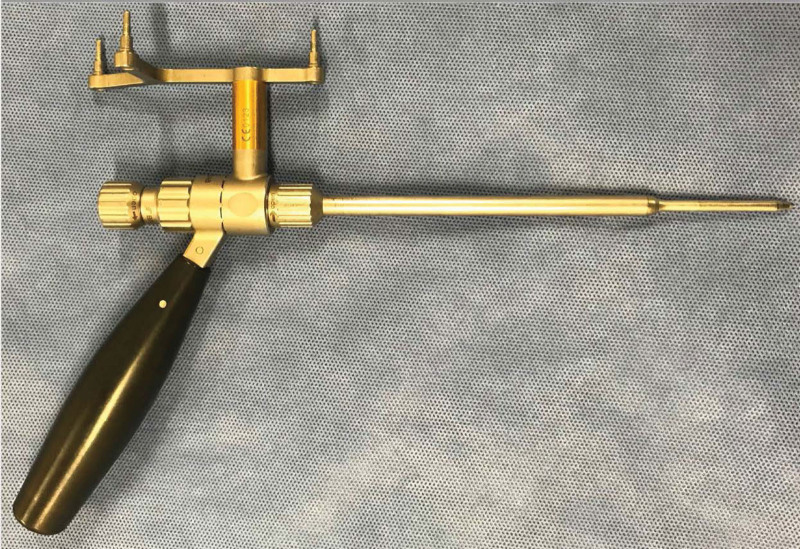
The calibrated target device (Brainlab: Munich, Germany).

Patients were administered general anesthesia and placed in the lateral decubitus or prone position on the surgical table. The patient in Group L was placed in the lateral decubitus position with a non-expandable bandage. The patients in group P were placed in a prone position on a Relton–Hall frame. For the 3D fluoroscopy-based navigation system, the reference frame was attached to the spinous process or posterior superior iliac spine, and Artis-Zee (Siemens, Munich, Germany) was positioned to obtain 3D fluoroscopic images, which were transferred to the Kick spine navigation system (Brainlab, Munich, Germany) for automatic registration. A guidewire inserted through the calibrated target device (Figs. 1 and 2; Brainlab, Munich, Germany) was used to position the PPS, assisted by the 3D fluoroscopy-based navigation system. The navigation system was used in the same manner, and the same technology was used in each group. After appropriately sized PPSs were placed, an intraoperative 3D fluoroscopic image scan including sagittal, axial, and coronal reconstruction images was obtained. An incorrectly placed PPS that could not be accepted was removed and reinserted.

### 2.3. Evaluation of accuracy for PPS insertion

**Figure 3.**
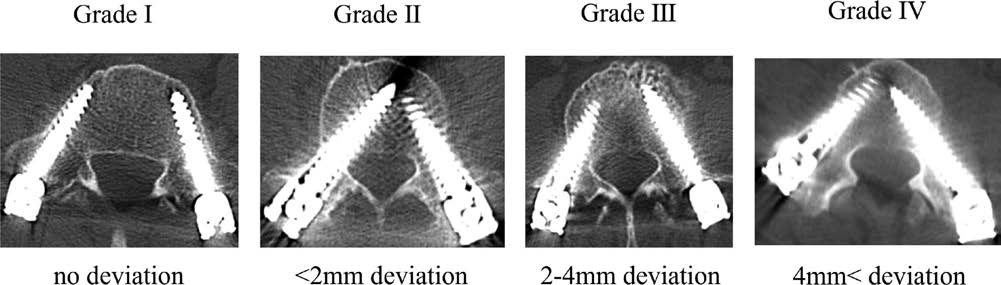
Ravi scale grades (Grade I: no deviation, Grade II: <2 mm, Grade III: 2–4 mm, Grade IV: >4 mm).^[7]^

PPS position was evaluated using postoperative computed tomography images. Three senior spine surgeons assessed the accuracy of PPS insertion according to Ravi scale grades (Grade I: no deviation, Grade II: <2 mm, Grade III:2–4 mm, Grade IV: >4 mm) and the direction of any deviation (cranial, caudal, medial, or lateral from the pedicle) (Fig. 3).^[7]^ All measurements were performed whereby kappa and alpha intra- and interrater reliability measurements noted as excellent (κ = 0.81–1.00) and good (κ = 0.61–0.80), respectively. If there was a difference in the grading between the 3 senior doctors, the grading with the highest value was adopted. When the PPS was reinserted, grading with the highest value was adopted. The longest distance between the vertebrae in which the PPS was placed and the reference frame for safe PPS insertion (LVR) were also compared between groups L and P.

### 2.4. Statistical analysis

Statistical analysis was performed using Statcel-the useful add-in software forms in Excel 4th ed. (OMS Publishing, Yanai H, Tokyo, Japan), and statistical significance was set at *P* < .05. The independent samples t-test was used for the expected normally distributed parameters, with data expressed as the mean ± SD (age and BMI). The chi-squared test was performed for qualitative data (sex, diagnosis, PPS deviation, deviation direction, and the relationship between the PPS deviation and position in relation to the reference frame).

## 3. Results

### 3.1. Demographic data

A total of 265 consecutive patients (group L: 78 patients; group P: 187 patients) were included in the analyses. There was a significant difference in the proportion of sex between the 2 groups (male, group L: 33 [42%], group P: 114 [60%]; *P* = .005), but the other patient-specific characteristics (age and BMI) showed no significant differences. The diagnostic variables of eligible patients were degenerative disease, trauma, tumor, and infection (169 [63.77%], 80 [30.19%], 9 [3.40%], and 7 [2.64%], respectively). The diagnostic variables of Group L were degenerative disease, trauma, tumor, and infection (49 [62.82%], 24 [30.77%], 3 [3.85%], and 2 [2.56%], respectively). The diagnostic variables of Group P were degenerative disease, trauma, tumor, and infection (120 [64.17%], 56 [29.95%], 6 [3.21%], and 5 [2.67%], respectively). There was no significant difference in the distribution of the diagnostic variables.

A total of 1816 PPSs were inserted in the thoracic spine (T1–T12) (n = 588) and lumbosacral spine (L1–S) (n = 1228) across both cohorts (Table 1). A total of 453 PPSs were inserted in Group L and 1363 PPS were inserted in Group P.

**Table 1:** Patient-specific characteristics and treatment-related characteristics.

	Total	Group L	Group P	*P* value
Number of patients	265	78	187	
Age (yr)	66.01 ± 13.35	65.21 ± 13.98	67.92 ± 11.25	.132
Sex				.005
Male	147 (55%)	33 (42%)	114 (60%)	
Female	118 (45%)	45 (58%)	63 (40%)	
BMI (kg/m^2^)	23.84 ± 8.70	23.79 ± 1.24	23.97 ± 4.35	.731
Diagnosis				
Degenerative disease (%)	169 (63.77)	49 (62.82)	120 (64.17)	.835
Trauma (%)	80 (30.19)	24 (30.77)	56 (29.95)	.894
Tumor (%)	9 (3.40)	3 (3.85)	6 (3.21)	.794
Infection (%)	7 (2.64)	2 (2.56)	5 (2.67)	.960
No. of thoracic spine screw	588	100	488	
No. of lumbosacral spine screw	1228	353	875	
No. of total screws	1816	453	1363	

BMI = body mass index.

### 3.2. PPS deviation and complications

In both cohorts, 1816 PPS were inserted, and the assessment based on Ravi scale found that a total of 76 PPS (4.18%) deviated (Grade II, III, or IV), with 21 (4.64%) of these in Group L and 55 (4.04%) in Group P. There were no significant differences in deviation rates between groups L and P (Table 2). The data on the direction of PPS deviation are presented in Table 2.

**Table 2:** Screw-specific accuracy.

	Total (n = 1816)	Group L (n = 453)	Group P (n = 1363)	*P* value
PPS related complications				
Radiculopathy due to PPS	1	1	0	
Spinal cord deficit due to PPS	1	0	1	
Screw deviation				
Grade I, n (%)	1740 (95.81)	432 (95.36)	1308 (95.96)	.580
Grade II, n (%)	41 (2.25)	11 (2.42)	30 (2.20)	.778
Grade III, n (%)	17 (0.94)	6 (1.32)	11 (0.81)	.322
Grade IV, n (%)	18 (0.99)	4 (0.88)	14 (1.03)	.788
Grade II, III, and IV, n (%)	76 (4.18)	21 (4.64)	55 (4.04)	.580
Grade III and IV, n (%)	35 (1.93)	10 (2.21)	25 (1.83)	.617
Direction of screw deviation				
Medial, n (%)	16 (21.05)	3 (14.29)	13 (23.64)	.371
Lateral, n (%)	55 (72.37)	18 (85.71)	37 (67.27)	.108
Cranial, n (%)	3 (3.95)	0	3 (5.45)	.275
Caudal, n (%)	1 (1.36)	0	1 (1.82)	.534
Cranial + Lateral, n (%)	1 (1.36)	0	1 (1.82)	.534

PPS = percutaneous pedicle screw.

One patient in group L experienced radiculopathy due to grade IV PPS deviation (lateral direction, L2 level). One patient in group P experienced a spinal cord injury due to a grade IV PPS deviation (medial direction, T7 level). No other PPS-related complications occurred in either of the groups.

### 3.3. Relationship between PPS deviation and distance from reference frame

In both groups, the distance between the PPS and reference frame was investigated. The distances between the PPS and reference frame in Groups L and P are listed in Table 3. Regarding LVR, 3 segments were detected in Group L (12.5% vs 4.04%; *P* = .028) (Table 4), while no obvious distance related to the PPS deviation was found in Group P (Table 4).

**Table 3:** The relation of the screw deviation and the distance between the screw and the position of the reference frame in Groups L and P.

	Grade I	Grade II	Grade III	Grade IV	Total	Deviation rate, %
Group L						
Same level	26	2	1	0	29	10.34%
1 segment	157	1	2	3	163	3.68%
2 segments	170	3	1	0	174	2.30%
3 segments	51	3	1	0	55	7.27%
4 segments	22	2	0	0	24	8.33%
5 segments	4	0	0	0	4	0%
6 segments	1	0	1	0	2	50%
7 segments	1	0	0	1	2	50%
Group L total	432	11	6	4	453	4.64%
Group P						
Same level	299	3	1	3	306	2.29%
1 segment	525	12	6	3	546	3.85%
2 segments	347	12	3	3	365	4.93%
3 segments	123	3	1	3	130	5.38%
4 segments	12	0	0	2	14	14.2%
5 segments	2	0	0	0	2	0%
Group P total	1308	30	11	14	1363	4.04%

**Table 4:** The relation of the screw-reference frame distance and screw deviation in group L and group P.

	≦3 group	≧4 group	*P* value
Ravi grades			
Grade II, III, and IV In Group L	18/421 (4.04%)	4/32 (12.5%)	.028
Grade II, III, and IV In Group P	53/1347 (3.93%)	2/16 (14.29%)	.083

### 3.4. PPS deviation for the upside or downside screw in Group L

In Group L, all PPS were designated as upside or downside PPS. A total of 223 upside PPSs were inserted, and 8 of which deviated (3.59%). A total of 230 downside PPS were inserted, and 13 of which deviated (5.65%). There was no significant difference in the deviation rate between the upside and downside PPS positions. There were 3 medial deviations and 5 lateral deviations in the upside PPS and 13 lateral deviations in the downside PPS (Table 5). The downside PPS was more likely to deviate in the lateral direction than the upside PPS (*P* = .017).

**Table 5:** Screw deviation focusing on the upside or downside screw in Group L.

	Upside screw (n = 223)	Downside screw (n = 230)	*P* value
Ravi scale grade Grade II, III, and IV	8 (3.59%)	13 (5.65%)	.296
Deviation direction			
Lateral	5 (62.5%)	13 (100%)	.017

## 4. Discussion

PPS insertion was improved by using an intraoperative 3D navigation system. Previous studies have shown the accuracy of PPS placement and clinical outcomes in the prone position;^[7–13]^ however, the accuracy of PPS placement in the lateral decubitus position remains unclear. Although there are some reports on the accuracy of PPS in the lateral decubitus position, most of them discuss the deviation rate in PPS insertion under fluoroscopic guidance, without the benefit of 3D fluoronavigation.^[3,5,6]^ Furthermore, we could not find any reports that compared the rate of PPS deviation between the prone and lateral decubitus positions for PPS insertion using 3D fluoronavigation in a single institution.

A previous study showed a deviation rate of 5.1% in the lateral decubitus position using fluoroscopy.^[6]^ Ouchida et al, however, found deviation rates of 1.8% in the lateral decubitus position and 4.0% in the prone position using O-arm-based navigation.^[4]^ In our study, the accuracy of PPS placement with 3D fluoroscopy-based navigation was similar between the lateral decubitus position (4.64%) and prone position (4.04%), which supports the findings of previous studies.^[4,6]^

Consensus on the usefulness of surgery in the lateral decubitus position has been reached in recent years through a series of publications. Ouchida et al reported that a single-position, minimally invasive, lateral decubitus position reduced the occupancy time of the operating room and workforce requirements.^[4]^ Ikuma et al reported successful treatment of thoracolumbar spinal fracture accompanied by diffuse idiopathic skeletal hyperostosis.^[14]^ Finally, Yamamoto et al reported a case of a patient with a unilateral and vertically unstable pelvic fracture treated with minimally invasive spinopelvic fixation, with retention of the anterior external fixator.^[15]^

Uehara et al reported that when including minor deviations, a distance of 3 vertebrae or more above or below the reference frame vertebra produces significantly more deviations^.[16]^ However, in that report, pedicle screws were inserted with an open technique in the prone position using matched navigation with preoperative computed tomography. In our study, in the lateral decubitus position, the distance of the 4 vertebrae between the screws and reference frame was a risk factor for PSS deviation. At our institution, the patient is held in the lateral decubitus position with only a nonexpandable bandage, which allows for more potential patient movement than in the prone position. While movement may help push the patient into a better position for screw placement, it makes navigation inaccurate. As this potential for movement could affect the accuracy of 3D fluoroscopy-based navigation, we recommend that this position be avoided for PPS across more than 4 vertebrae.

In our study, the direction of the downside PPS deviation tended to be toward the lateral side. This could be due to space restrictions between the patient spine and the surgical table, which is considered a major problem with this technique. The surgeon cannot get their hand enough in the lateral to medial direction to use a normal screw trajectory because of the space restriction from the patient to the surgical table in the lateral decubitus position. Hiyama et al reported difficulty accessing the downside PPS insertion angle with the patient in the lateral decubitus position because of the limited working space between the operative table and fluoroscopy instrumentation, but they overcame this using the Viper Prime (DepuySynthes Spine, Inc., Massachusetts), which is known for the integration of the screw and stylet,^[5]^ and We think some tools similar to this may be beneficial in ameliorating the risk of deviations in downside screws.

The limitations of this study are its retrospective cohort design, all PPS surgeries were performed by 3 surgeons at a single institute, and the fact that radiological analysis was performed by 2 physicians who coauthored this report. A total of 265 patients with 1816 PPS constituted a relatively small sample size, and bias was inevitable. Additionally, the underlying pathologies were heterogeneous, which could have affected several parameters and the quality of the study. There was also a predominance of males in Group P; however, previous reports have provided no evidence that sex has any effect on screw deviation rates. Therefore, we believe that differences in sex distribution did not affect the results.

In conclusion, there was no significant difference in PPS deviation regardless of the intraoperative patient positioning when a 3D fluoroscopy-based navigation system was utilized for PPS insertion. Our results suggest that the safety and efficacy of PPS insertion in the lateral decubitus position is similar to that in the conventional prone position. However, surgeons should keep in mind that it is easy to deviate toward the lateral side of the downside PPS.

## Author contributions

**Resources:** Tomohiro Inoue, Masataka Ueda, Tomohiko Hirose, Kazutoshi Otsuka, Keisuke Kawasaki.

**Writing – original draft:** Ryuichiro Okuda.

**Writing – review & editing:** Hisanori Ikuma.
